# Tumor-associated characteristics and immune dysregulation in nasopharyngeal carcinoma under the regulation of m7G-related tumor microenvironment cells

**DOI:** 10.1186/s12957-024-03441-2

**Published:** 2024-06-25

**Authors:** Zhen Long, Xiaochen Li, Wenmin Deng, Yan Tan, Jie Liu

**Affiliations:** 1 Department of Otorhinolaryngology Head and Neck Surgery, The Sixth Affiliated Hospital, Sun Yat-sen University, No. 26, Yuancun Erheng Road, Tianhe District, Guangzhou City, Guangdong Province China; 2 Biomedical Innovation Center, The Sixth Affiliated Hospital, Sun Yat-sen University, Guangzhou, China

**Keywords:** Nasopharyngeal carcinoma, m7G, Tumor microenvironment, Immune cell, Fibroblast

## Abstract

**Background:**

Nasopharyngeal carcinoma (NPC) is a type of malignant tumor with high morbidity. Aberrant levels of N7-methylguanosine (m7G) are closely associated with tumor progression. However, the characteristics of the tumor microenvironment (TME) in NPC associated with m7G modification remain unclear.

**Methods:**

A total of 68,795 single cells from single-cell RNA sequencing data derived from 11 NPC tumor samples and 3 nasopharyngeal lymphatic hyperplasia (NLH) samples were clustered using a nonnegative matrix factorization algorithm according to 61 m7G RNA modification regulators.

**Results:**

The m7G regulators were found differential expression in the TME cells of NPC, and most m7G-related immune cell clusters in NPC tissues had a higher abundance compared to non-NPC tissues. Specifically, m7G scores in the CD4^+^ and CD8^+^ T cell clusters were significantly lower in NPC than in NLH. T cell clusters differentially expressed immune co-stimulators and co-inhibitors. Macrophage clusters differentially expressed EIF4A1, and high EIF4A1 expression was associated with poor survival in patients with head and neck squamous carcinoma. EIF4A1 was upregulated in NPC tissues compared to the non-NPC tissues and mainly expressed in CD86^+^ macrophages. Moreover, B cell clusters exhibited tumor biological characteristics under the regulation of m7G-related genes in NPC. The fibroblast clusters interacted with the above immune cell clusters and enriched tumor biological pathways, such as FGER2 signaling pathway. Importantly, there were correlations and interactions through various ligand-receptor links among epithelial cells and m7G-related TME cell clusters.

**Conclusion:**

Our study revealed tumor-associated characteristics and immune dysregulation in the NPC microenvironment under the regulation of m7G-related TME cells. These results demonstrated the underlying regulatory roles of m7G in NPC.

**Supplementary Information:**

The online version contains supplementary material available at 10.1186/s12957-024-03441-2.

## Introduction

Nasopharyngeal carcinoma (NPC) is a malignant tumor that originates from the epithelial lining of the nasopharyngeal mucosa and includes nodular lesions, ulcerative lesions, and submucosal infiltration [1]. The incidence of NPC is mainly concentrated in East and Southeast Asia, of which China has the largest number of cases, accounting for 47.7% of cases worldwide [2]. The interactive nature of genetic susceptibility and Epstein-Barr virus (EBV) infection is the leading cause of NPC pathogenesis [3]. The intrinsic collaboration of the tumor microenvironment (TME), TME cells, and EBV are the key factors that promote NPC pathogenesis and immune escape [4]. Clinical observations have revealed that a substantial number of stromal and immune cells are often interspersed with malignantly transformed nasopharyngeal epithelial cells. However, a comprehensive analysis of the NPC TME is still unclear.

The TME affects the onset, growth, invasion, and anti-immune effects of tumors through a variety of mechanisms, such as immunoassay sites, and strengthening or modifying T cells (chimeric antigen receptor CAR-T) [5, 6]. Previous NPC studies have focused on the functional roles of TME cells. For example, NPC cells with high expression of CD70^+^ serve as the starting gate of lipid metabolism, controlling the development of lipid activity and homeostasis of regulatory T cells, and providing opportunities for immune escape [7]. Previously, a tissue biopsy showed that infiltrated cells in NPC were mixed with lymphocytes, stromal cells, and tumor-associated cells of heterogeneous shape [8]. In terms of NPC, a study has found crosstalk and metastasis signals of tumor-associated macrophages (TAM) [9]. The discoveries of M2-TAM polarization-related regulatory mechanisms revealed ZIC2, JUNB, and CD163 as therapeutic targets for NPC immunotherapy [10]. Cancer-associated fibroblasts (CAFs) promote the survival of irradiated NPC cells via the NF-kappa B (NF-κB) [11]. Activation of YAP1/FAPα CAFs was induced by EBV viral products containing exosomes and provided a favorable microenvironment for NPC progression [12]. This evidence implies the multidimensional function of TME cells in NPC.

N7-methylguanosine (m7G) is a novel posttranslational modification that is usually found in the internal modification of mRNA [13], transfer RNA, rRNA, and lncRNA in mammals [14]. The m7G regulators, such as METTL1, WDR4, AGO2, and NUDT1, can be considered cancer biomarkers, and m7G modification frequently correlates to tumorous prognosis in certain cancers, including intrahepatic cholangiocarcinoma [15], bladder cancer [16], uterine corpus endometrial carcinoma [17], gastric cancer [18], and hepatocellular carcinoma [19]. In recent years, few studies have demonstrated the regulatory roles of m7G modification for NPC. For instance, METTL1/WDR4 promotes NPC growth and metastasis by upregulating the WNT/β-catenin signaling pathway and promoting epithelial-mesenchymal transition (EMT) and chemoresistance to cisplatin and docetaxel [20]. However, an overview of m7G modification regulators in the NPC TME has not been clearly presented.

Given the individual heterogeneity and complexity of m7G modification, TME cells from single-cell RNA sequencing (scRNA-seq) data were clustered based on the expression of m7G regulators to investigate that whether m7G regulators affected the TME of NPC. Our results provided an overview of the expression of m7G regulators in the NPC microenvironment and revealed immune dysregulation mediated by m7G modification. Our findings highlighted the crucial roles of m7G regulators in the NPC microenvironment and provided a new foundation for precision in NPC therapy.

## Methods

### Visualization of TME cell types in NPC

We collected scRNA-seq data from nasopharyngeal tumor tissues in 11 patients with NPC and nasopharyngeal lymphatic hyperplasia (NLH) samples in 3 patients with NLH in the GSE150825 project, and a total of 68,795 cells were obtained. The Seurat package in R software (version 4.3.0.1) was used to perform the quality control. Our screening criteria were as follows: (1) the number of genes in a single cell was > 200 and < 4000; (2) the proportion of mitochondria was < 15%; and (3) DoubletFinder software was used to recognize doublets, which were removed. Principal component analysis and uniform manifold approximation and projection (UMAP) method were then used for dimensionality reduction and clustering. We annotated the qualified cells using the Blueprint Encode Data dataset in the Single R package and the celldex package, followed by correction with known marker genes.

### Nonnegative matrix factorization (NMF) algorithm clustered the m7G-related TME cells

According to previous studies, a total of 62 m7G-related genes have been didentified, and 61 m7G-related genes were expressed in the NPC TME cells. The NMF R package was applied to cluster the NPC TME cells based on the expression of the 61 m7G regulators to generate m7G-related subtypes. Gene set variation analysis (GSVA) in R software and the ssGSEA algorithm were used to calculate m7G scores for these NMF clusters. The m7G scores of the individual NMF clusters were presented as box plots and violin plots produced using the ggplot2 package.

### Analysis of cell–cell communication and immune cell infiltration

The GSE68799 dataset was downloaded from the Gene Expression Omnibus (GEO) database, and Cibersort software was used to analyze the infiltration levels of the immune cell clusters. Cell Chat package in R software was applied to recognize and calculated the cell–cell communication among the various cell clusters.

### Functional enrichment analysis and single-cell regulatory network inference and clustering (SCENIC) analysis of NMF m7G-related clusters

Cluster Profiler in R software was used to determine the enriched signaling pathways in the Reactome database for the m7G-related NMF clusters. GSVA package was used to analyze the pathway enrichment of hallmark genes in NMF clusters. SCENIC (version 0.12.0) was applied to investigate the gene regulatory network of the transcription factors (TFs) in NPC.

### Distinctive features in NMF m7G-related clusters of T cells were evaluated

Function signatures of T cells (exhaustion, cytotoxic, effector, and evasion) were scored using the GSVA package. The average expression of immune stimulators, inhibitors, and T cell function marker genes was calculated. Then, Complex Heatmap package was generated to draw the levels of distinctive features in NMF m7G-related T cell clusters.

### Survival analysis

We used Gene Expression Profiling Interactive Analysis to identify the expression levels of the m7G-related genes in head and neck squamous cell carcinoma (HNSCC). Additionally, we downloaded the GSE102349 [21] dataset from the GEO database to acquire bulk public RNA‑seq datasets, which contained 113 tumor samples from patients with NPC. According to gene expression profiling from the bulk RNA‑seq datasets, the xCell package in R software was used to calculate the fibroblast proportion in each NPC sample. These samples were further divided into high and low groups based on their fibroblast proportion. Then, the survminer in R package was used to establish Kaplan-Meier curves.

### Immunofluorescence

NPC samples and their paracancerous tissues (adjacent non-NPC tissues) were collected from participants and used for immunofluorescence staining. Paraffin sections were routinely deparaffinized, hydrated, antigens retrieved, and blocked. Each sample was repeated in triplicate. The sections were then incubated with a mouse anti-EIF4A1 antibody (1:200 dilution, HY-P80650, MCE), a rabbit anti-CD86 antibody (1:200 dilution, ab239075, Abcam), and a rabbit anti-163 antibody (1:200 dilution, ab182422, Abcam) for 30 min at room temperature in the dark. The slides were then incubated using a horseradish peroxide-conjugated secondary Ms + Rb antibody (1:2000 dilution, B001, Ebiogo) for 30 min at room temperature in the dark. Images were captured after nuclear staining with DAPI using a digital slide scanner (Pannoramic MIDI, 3DHISTECH). This project was approved by the Ethics Committee of The Sixth Affiliated Hospital, Sun Yat-sen University. All informed consents of patients were acquired.

## Results

### Landscape of the microenvironment and m7G regulators in NPC

**Fig. 1:**
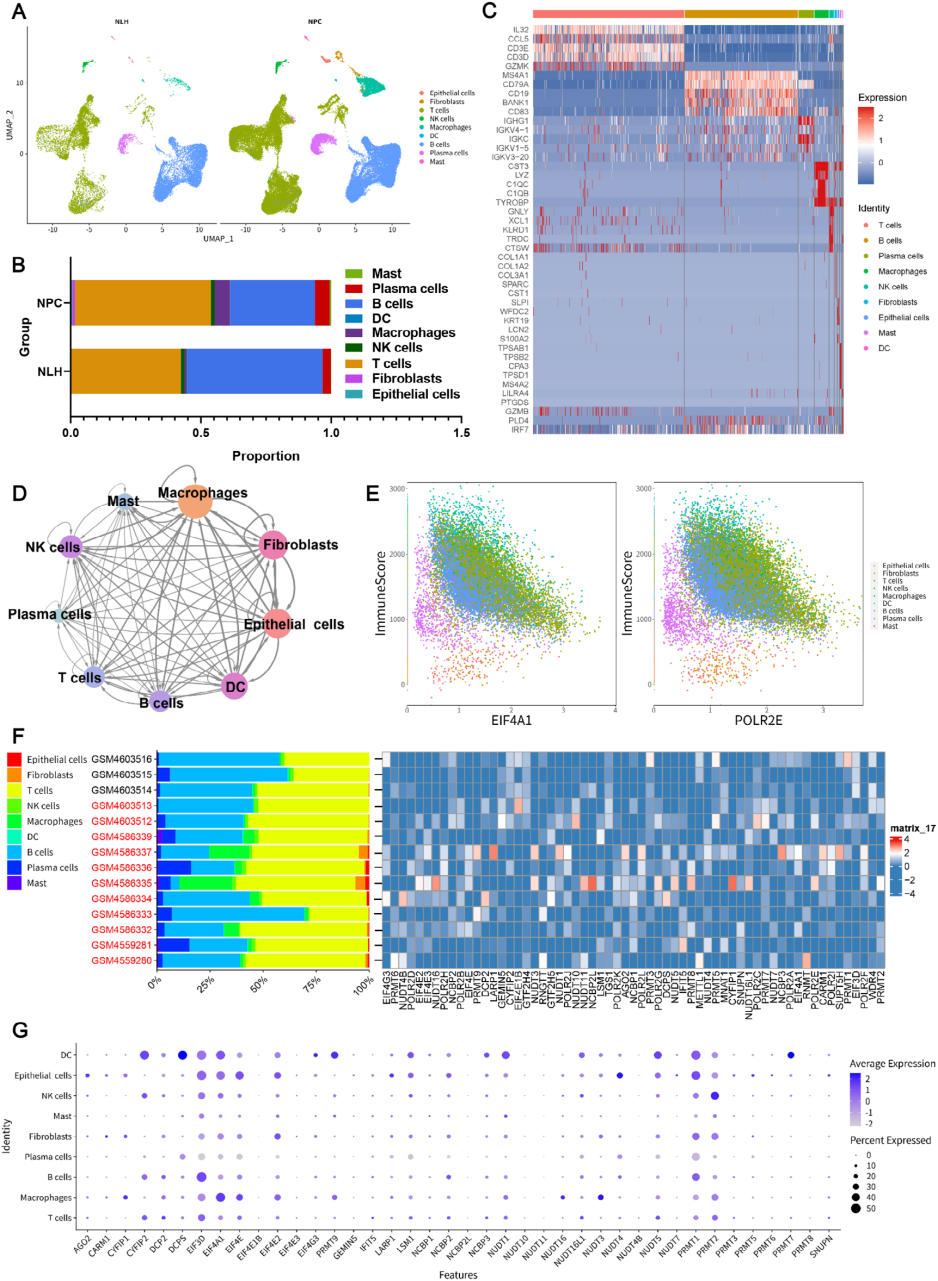
Overview of cells in the tumor microenvironment (TME) in the single-cell data for nasopharyngeal carcinoma (NPC). **A** UMAP plot showed the composition of the 9 major cell types in the TME of NPC and nasopharyngeal lymphatic hyperplasia (NLH). **B** Bar graph showing the percentage of the 9 major cell types in the NPC and NLH groups. **C** Heatmap of the differentially expressed genes among the 9 cell types. **D** Crosstalk between the 9 cell types was determined by cell–cell communication analysis. **E** Scatter plots indicated the association of ImmuneScore with the m7G-related genes EIF4A1 and POLR2E. **F** The cell type distributions (left bar graph) and expression profiles of the m7G regulators (right heatmap) in NPC (marked with red words) and NLH samples are shown. **G** Bubble plot depicting the expression of m7G-related genes in the main 9 cell types. The average expression of genes is marked in different colors, and the expressed level percentage is represented by bubble size

The Seurat package was used for the quality control of all cells from scRNA-seq data, and a total of 68,795 cells were retained, including 17,018 cells from the 3 NLH samples and 51,777 cells from the 11 NPC samples (Supplementary Fig. S1A). The qualified cells were clustered into 9 major cell types, including epithelial cells, fibroblasts, T cells, natural killer (NK) cells, macrophages, dendric cells (DC), B cells, plasma cells, and mast cells (Fig. 1A). Among the 9 TME cell types, T cells and B cells were the most predominant cell types in the NPC microenvironment, and fibroblasts, macrophages, and plasma cells had a higher percentage in the NPC samples than in the NLH sample (Fig. 1B). The top five most differentially expressed genes (DEGs) in the 9 main cell types were presented in Fig. 1C. Moreover, cell–cell communication analysis showed diverse interactions among these cell types, with a higher frequency of interactions between macrophages and epithelial cells, fibroblasts, and DCs (Fig. 1D and Supplementary Fig. 1B). Compared to the NLH group, the NPC group had more upregulated differentially expressed genes (Supplementary Fig. 1C), which were enriched in the viral process-associated Gene Ontology (GO) terms, such as regulation of viral life cycle, regulation of viral process, and response to virus (Supplementary Fig. S1D). These also included Kyoto Encyclopedia of Genes and Genomes (KEGG) pathways, such as NF-κB signaling pathway, Toll-like receptor (TLR) signaling pathway, NK cell-mediated cytotoxicity, and EBV infection (Supplementary Fig. S1E), which suggests a strong antiviral response in patients with NPC.

Among the 9 TME cell types, we found different strong associations between m7G regulators and ImmuneScore (Fig. 1E and Supplementary Fig. S1F). We also assessed the expression levels of m7G regulators in all samples. As expected, the 61 m7G regulators were differentially expressed in the 14 samples (Fig. 1F), and the 9 major cell types exhibited distinct expression profiles of m7G regulators (Fig. 1G). Taken together, these results indicate the differential expression of m7G regulators in the NPC microenvironment.

### M7G-related immune cell clusters displayed different infiltration levels and were correlated with each other

**Fig. 2:**
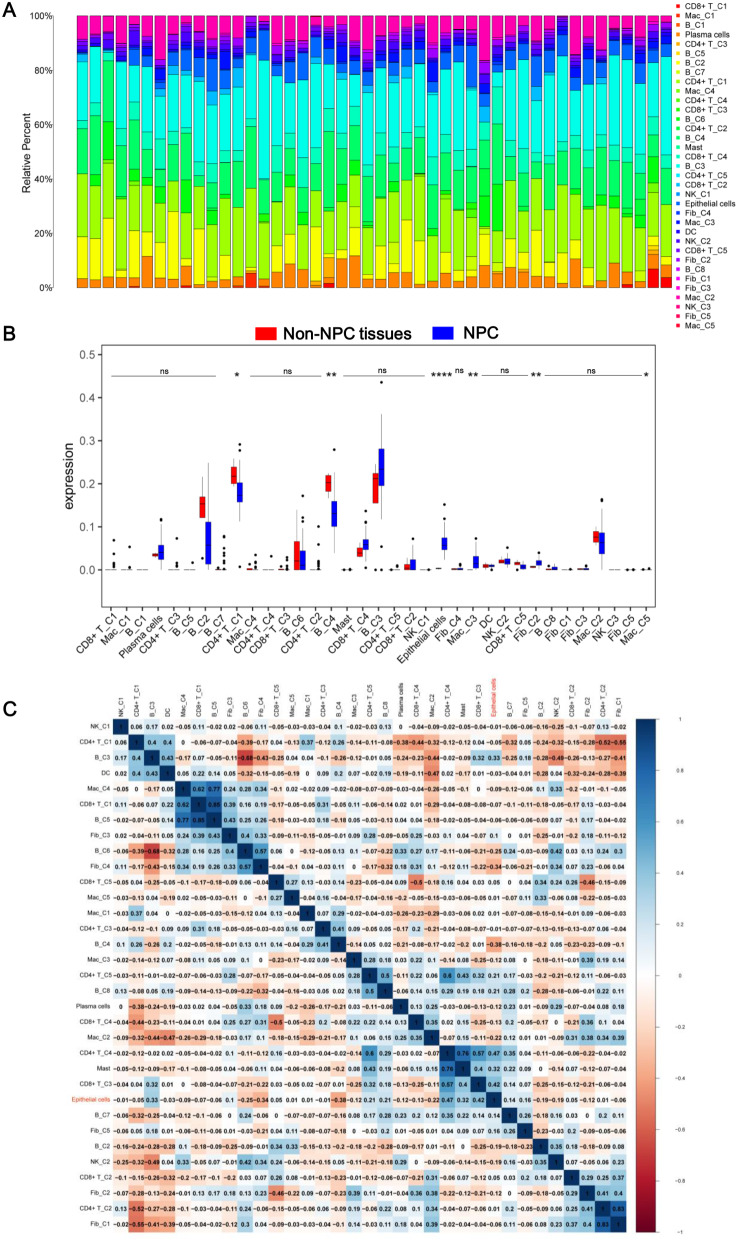
Immune infiltration analysis of the GSE68799 dataset. **A** Infiltration of immune cell clusters in the GSE68799 dataset. **B** Box plot showing the levels of immune cell clusters in non-nasopharyngeal carcinoma (non-NPC) tissues and NPC tissue samples. **C** Heatmap depicting the correlations between the NMF immune cell clusters. The blue and red colors represent positive and negative correlations, respectively. ***p < 0.001; **p < 0.01; *p < 0.05; ns, no significance

Based on the expression of m7G-related genes, immune cells were further clustered using the NMF algorithm, which included CD4^+^ T cell C1-C5 clusters (CD4^+^ T_C1-C5), CD8^+^ T cell C1-C5 clusters (CD8^+^ T_C1-C5), macrophage C1-C5 clusters (Mac_C1-C5), B cell C1-C8 clusters (B_C1-C8), and NK cell C1-C3 clusters (NK_C1-C3). Subsequently, we utilized the GSE68779 dataset to analyze the infiltration of m7G-related immune cell clusters in the NPC TME, as shown in Fig. 2A. Notably, the infiltrated levels of CD4^+^ T_C2, B_C3, and Mac_C3 in tumor samples were significantly higher than that in normal samples (Fig. 2B). By analyzing the correlation between immune cells, we observed that CD4^+^ T_C1 was positively and negatively correlated with B_C3 and CD4^+^ T_C2, respectively (Fig. 2C). These findings revealed the infiltration levels of m7G-related immune cell clusters and the different correlations between these clusters in the NPC microenvironment.

### m7G-related T cell clusters exhibited unique characteristics of immune stimulation and inhibition in the NPC microenvironment

**Fig. 3:**
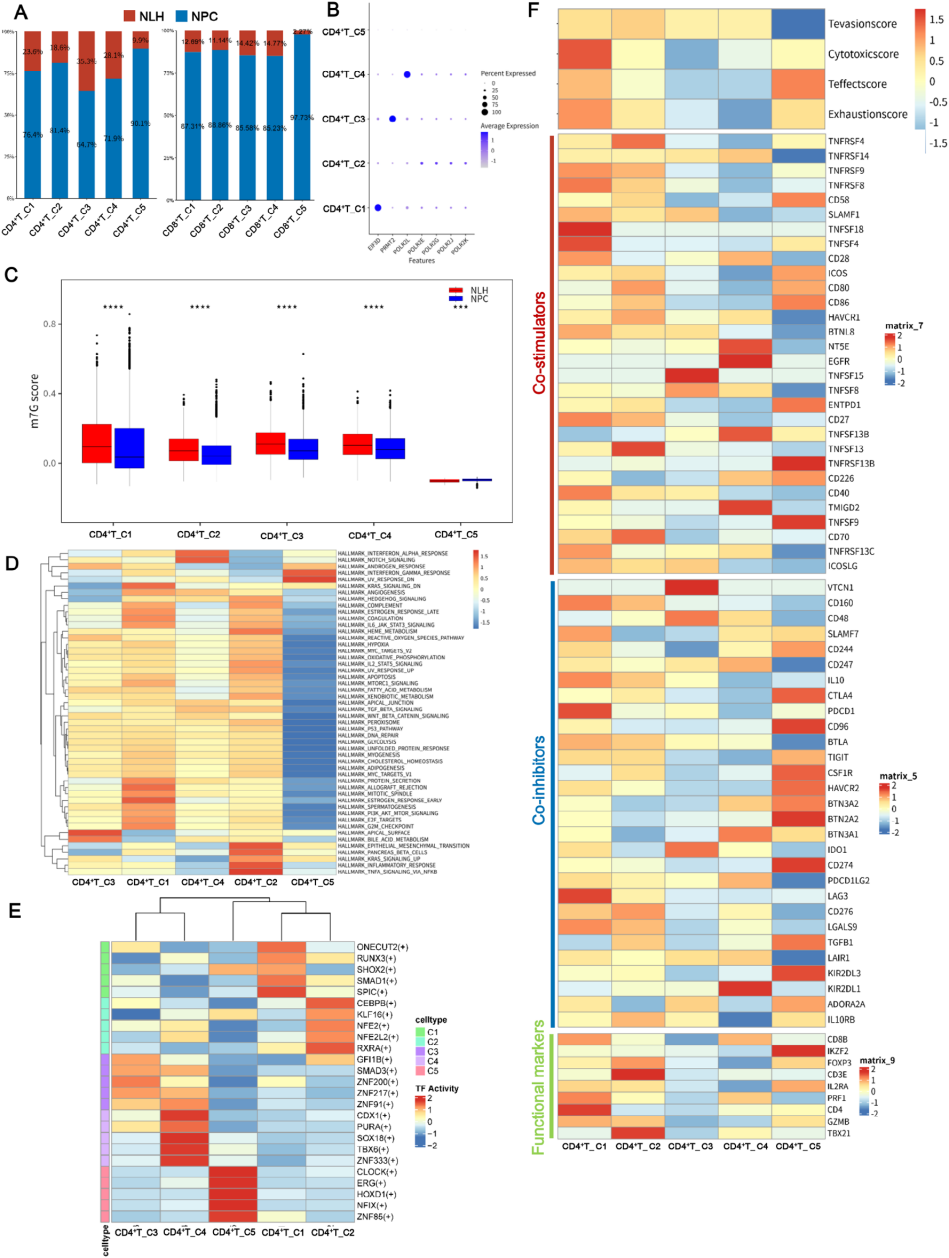
M7G-related T cell clusters exhibited characteristics of immune inhibition and immunosuppression. **A** Bar graph depicting the percentage of the CD4^+^ and CD8^+^ T cell clusters in nasopharyngeal carcinoma (NPC) and nasopharyngeal lymphatic hyperplasia (NLH) samples. **B** The different expression levels of m7G-related genes in CD4^+^ T cell clusters are illustrated by a bubble plot. **C** The m7G scores in nonnegative matrix factorization (NMF) CD4^+^ T cell clusters in NPC and NLH samples were calculated by GSVA. ****p < 0.0001; ***p < 0.001. **D** Heatmap of the hallmark pathway in CD4^+^ T cell clusters. **E** TFs were differentially activated in CD4^+^ T cell clusters. **F** Heatmap of T cell features among m7G-related CD4^+^ T clusters, showing the T exhaustion, T cytotoxic, T effector, and T evasion scores, as well as the expression levels of immune stimulators, inhibitors, and T cell function signature genes in the CD4^+^ T clusters

T cells play a vital role in antitumor immunity. We observed that each m7G-related CD4^+^ and CD8^+^ T cell cluster had a higher percentage in NPC samples than in NLH samples (Fig. 3A). The expression levels of the different m7G regulators in CD4^+^ and CD8^+^ T cell clusters were shown in Fig. 3B and Supplementary Fig. S2A, and we noted that eukaryotic translation factors, such as EIF3D, and RNA polymerase subunits, such as POLR2L, POLR2G, and POLR2E, were differentially expressed in the CD4^+^ and CD8^+^ T cell clusters. GSVA was used to assess the m7G score in the CD4^+^ and CD8^+^ T cell clusters based on the expression of m7G-related genes. We observed that the m7G scores of several clusters in the NPC group were significantly decreased compared to those in the NLH group, such as CD4^+^ T_C1, CD4^+^ T_C2, and CD4^+^ T_C3 (Fig. 3C and Supplementary Fig. S2B). We next performed an enrichment analysis to explore the characteristics of these m7G-related T cell clusters. The generated heatmap showed that the hallmark pathways in CD4^+^ T_C2 were activated in the EMT and TNFA signaling pathways via NF-κB, which promotes tumor progression (Fig. 3D). SOX18, one of the SOX family members that is positively related to CD4^+^ T cells [22] and promotes regulatory T cell infiltration [23], was activated in CD4^+^ T_C4 (Fig. 3E). The TFs activities of the CD8^+^ T cell clusters were shown in Supplementary Fig. 2C. The overall effects of m7G-related genes on the T cell clusters were also assessed, and we found several differences in the average expression of stimulator, inhibitor, and function-related marker genes (Fig. 3F and Supplementary Fig. S2D). Notably, CD4^+^ T_C1 displayed higher cytotoxic score than the other CD4^+^ T clusters (Fig. 3F). Collectively, m7G-related T cell clusters exhibited differences in immune stimulation and inhibition in the TME.

### M7G-related macrophage clusters in the TME had distinct functional differentiation in the immune response

**Fig. 4:**
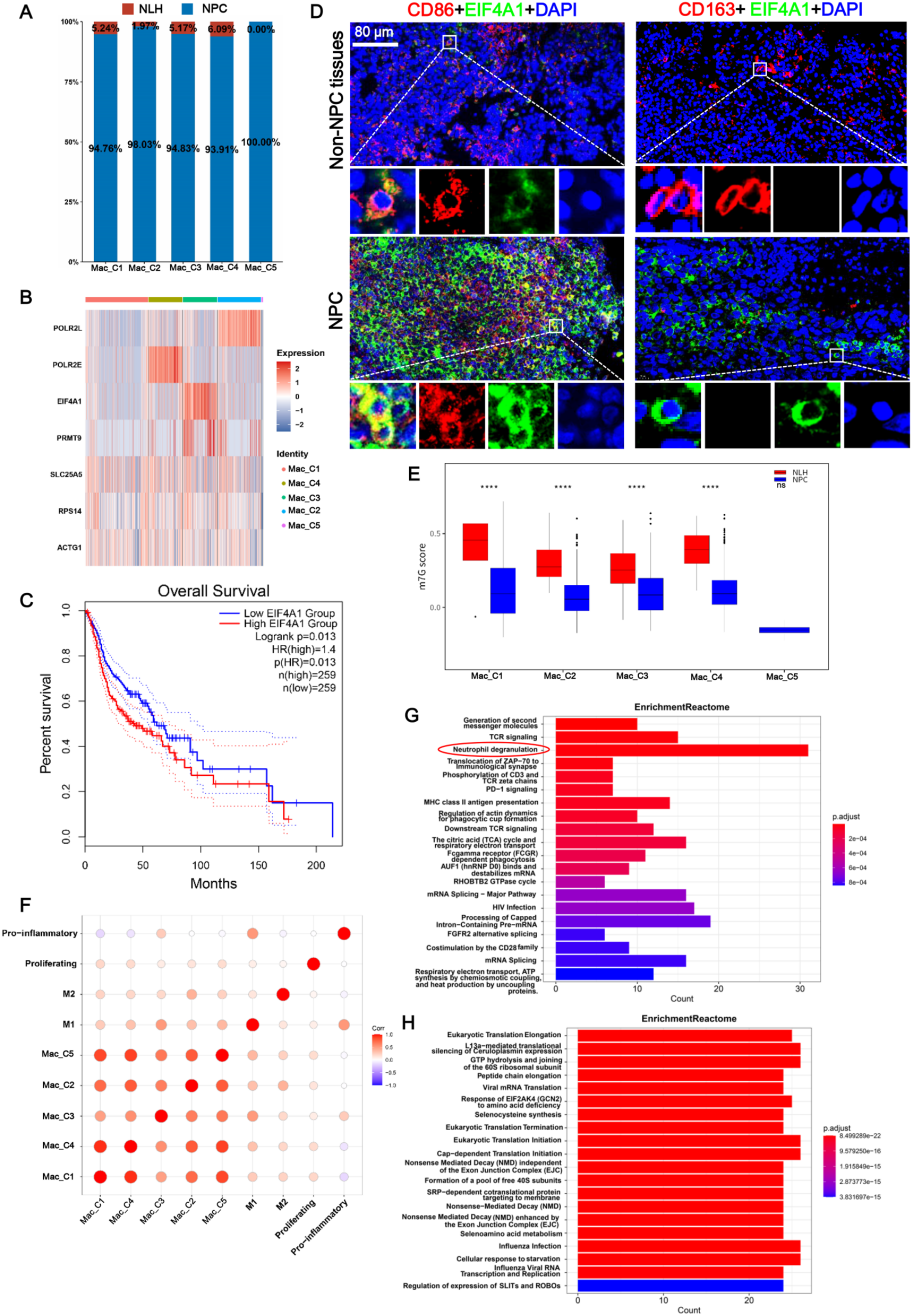
Nonnegative matrix factorization (NMF) clusters of macrophages exhibited heterogeneity in immune response and tumorigenesis. **A** Bar graph depicting the percentage of the five macrophage clusters in nasopharyngeal carcinoma (NPC) and nasopharyngeal lymphatic hyperplasia (NLH) samples. **B** Heatmap indicating the different expressions of m7G-related genes in the NMF macrophage clusters. **C** Kaplan-Meier survival analysis revealed that patients with head and neck squamous cell carcinoma (HNSCC) and low expression of EIF4A1 have better survival than those with high expression of EIF4A1. **D** Double immunofluorescence of NPC tissues and their adjacent paracancerous tissues (non-NPC tissues) revealed EIF4A1 expression in CD86^+^ and CD163^+^ macrophages (*n* = 3). DAPI was used to stain the cell nucleus. **E** The m7G scores in each macrophage cluster in the NPC and NLH groups are shown in the Box plot. ****p < 0.0001; ns, no significance. **F** Bubble plot indicating the correlations of the gene signatures of pro-inflammation, proliferating, M1, and M2 with the macrophage clusters. Red represents a positive correlation, and blue represents a negative correlation. **G**-**H** Reactome analysis of Mac_C1 (**G**) and Mac_C3 (**H**)

Macrophages are one of the most abundant cell types in the TME and exhibit different phenotypes and functions in response to various signals generated by TME cells [24]. Thus, we explored the phenotypes and functions of the m7G-related macrophage clusters in NPC. Our results revealed that the proportion of m7G-related macrophage C1-C4 clusters was increased in the NPC group compared to the NLH group (Fig. 4A). In addition, the m7G-related genes POLR2L, EIF4A1, and POLR2E were abundantly expressed in Mac_C2, Mac_C3, Mac_C4, respectively (Fig. 4B). Notably, the high expression of EIF4A1 was negatively correlated with survival in patients with HNSCC (Fig. 4C), and there were no significant differences between expression levels of POLR2L and POLR2E and HNSCC patient survival (Supplementary Fig. S3A). Thus, we performed double immunofluorescence to detect EIF4A1 expression in CD86^+^ and CD163^+^ macrophages in NPC tissues. The upregulation of EIF4A1 in NPC samples compared to non-NPC tissues presented in Fig. 4D, and a predominant co-localization with CD86^+^ macrophages rather than CD163^+^ macrophages was observed. Moreover, macrophage C1-C4 clusters in NPC samples exhibited lower m7G scores than in NLH samples (Fig. 4E). Next, the correlation between the macrophage signatures of pro-inflammation, proliferation, M1, M2, and the m7G-related macrophage clusters was evaluated using Pearson analysis (Fig. 4F). We found Mac_M2 and Mac_M3 exhibited a most positive correlation with M2 and M1 macrophages, respectively (Fig. 4F). In addition, Mac_C1 was most positively related to Mac_C4, and both Mac_C1 and Mac_C4 were negatively correlated with pro-inflammatory macrophages, which was in contrast with Mac_C3 (Fig. 4F). The generated heatmap of the hallmark pathway also showed an enriched inflammatory response in Mac_C3 (Supplementary Fig. S3B). Moreover, ARID3A and VDR, which participate in macrophage polarization [25, 26], were specifically activated in Mac_C3 (Supplementary Fig. S3C). In summary, the m7G-regulated macrophage clusters displayed different macrophage signatures, in which Mac_C3 exhibited an inflammatory signature.

To further investigate the relationship between m7G-related macrophage clusters and special pathways, we performed Reactome enrichment analysis of the m7G-related macrophages. We observed that Mac_C1 enriched the T cell receptor (TCR) signaling pathways (such as TCR signaling, phosphorylation of CD3 and TCR zeta chains, and downstream TCR signaling), MHC class II antigen presentation, Fc gamma receptor dependent phagocytosis, co-stimulation by the CD28 family, as well as PD-1 signaling (Fig. 4G), which could promote immune escape of the tumor cells [27]. This implicates that Mac_C1 was activated in multiple immune response pathways and was involved in the regulation of immune responses in T cells. TLR signaling pathways were abundantly accumulated in Mac_C2 (Supplementary Fig. S3D), such as MyD88 deficiency (TLR2/4), IRAK4 deficiency (TLR2/4), regulation of TLR by endogenous ligand, and disease associated with the TLR signaling cascade, which were related to the inflammatory mechanisms of in cancers [28, 29]. Interestingly, eukaryotic translation-related pathways were accumulated in Mac_C3, including eukaryotic translation elongation, eukaryotic translation initiation, and eukaryotic translation termination (Fig. 4H). The expression of SLIT and ROBO has a biological importance in mediating NPC cell migration [30]. We noted that Mac_C4 was involved in enriched TCR signaling-associated pathways and programmed cell death, as well as signaling by ROBO receptors and the regulation of the expression of SLITs and ROBOs, which had a biological function in mediating NPC cell migration [30] (Supplementary Fig. S3E). Notably, neutrophil degranulation was significantly enriched in Mac_C1, Mac_C2, and Mac_C4, indicating a close relationship between m7G-related macrophages and neutrophils (Fig. 4G and Supplementary Fig. S3D-S3E). To further explore the association between m7G-related macrophages and cancers, KEGG analysis was performed and its result revealed that m7G-related macrophage clusters, including Mac_C1-C4, were linked to multiple cancers, enhanced apoptosis, glycolysis/gluconeogenesis, and autophagy regulation (Supplementary Fig. S3F). Overall, m7G-related macrophage clusters might be involved in the regulation of the NPC microenvironment through immune response, eukaryotic translation, and NPC cells.

### B cell clusters regulated by m7G genes exhibited tumor-related characteristics

**Fig. 5:**
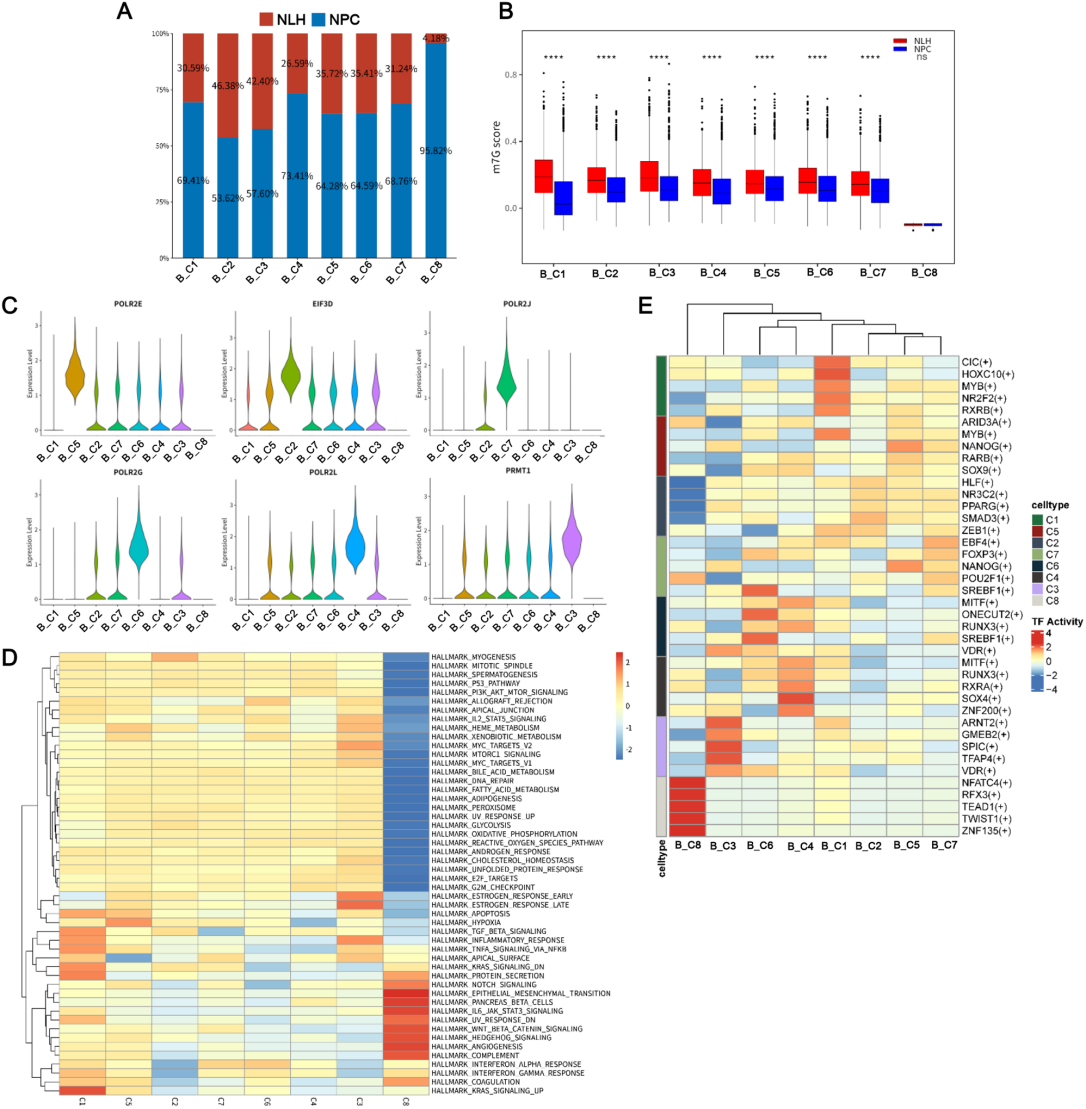
m7G-related B cells were associated with tumorigenesis. **A** Bar graph depicting the percentage of nonnegative matrix factorization (NMF) B cell clusters in nasopharyngeal carcinoma (NPC) and nasopharyngeal lymphatic hyperplasia (NLH) samples. **B** Box plot illustrating the m7G scores in each B cell cluster in the NPC and NLH groups. ****p < 0.001; ns, no significance. **C** The m7G regulators were differentially expressed in B cell clusters, as shown in the violin plots. **D** Gene set variation analysis (GSVA) shows the hallmark pathway enrichment in B cell clusters. **E** Heatmap of the transcription factor (TFs) activation patterns in the B cell clusters

The m7G-related B cells were identified into 8 clusters by NMF clustering, and B_C5 was the most expanded population in the NPC group compared to the NLH group (Fig. 5A). Among these clusters, the m7G scores of B_C1-C7 were significantly higher in the NLH group compared to the NPC group, whereas the *p* value of B_C8 exhibited no significant difference between the NLH and NPC groups (Fig. 5B). Several m7G regulators were differentially expressed in the B cell clusters, where PRMT1 was abundantly expressed in B_C3 (Fig. 5C). We performed GSVA and SCENIC to investigate the differences in function and transcriptional patterns. Interestingly, the regulatory pattern of the hallmark pathways of B_C8 was different from that of B_C1-C7, which were activated in multiple pathways, including the PI3K/AKT/MTOR signaling, glycolysis, hypoxia, and TGF beta signaling pathways (Fig. 5D). Furthermore, we found that B_C3 was enriched in the MYC targets V1/V2 (Fig. 5D), which is linked to NPC tumorigenesis [31]. TFs promoting NPC cell proliferation, migration and invasion, and EMT progression, such as SREBF1 [32], SOX9 [33], and SOX4 [34], were significantly activated in the m7G-related B cell clusters (Fig. 5E). Together, the above findings reflect the tumor-related characteristics of B cell clusters that were associated with NPC tumors.

### Functional analysis of m7G-related NK cells in the TME

The m7G-related NK cells were divided into C1-C3 clusters based on the expression of m7G-associated genes. The m7G score of NK_C1 was significantly higher in the NLH group than in the NPC group, and the m7G score of NK_C3 was < 0 in both groups (Supplementary Fig. S4A). In addition, the activation patterns of TFs among these clusters differed (Supplementary Fig. S4B). Interestingly, the hallmark pathways related to immune response (interferon alpha/gamma response, TNFA signaling via NF-κB, and inflammatory response), cell development (mitotic spindle, p53 pathway, PI3K/AKT/MTOR signaling, DNA repair, E2F targets, and G2M checkpoint), and cell metabolism (HEME metabolism, bile acid metabolism, fatty acid metabolism, adipogenesis, and glycolysis) were activated in NK_C1 and NK_C2, but were suppressed in NK_C3 (Supplementary Fig. S4C), which suggests the effects of the m7G-related genes on NK cell development and immune response.

### M7G-related fibroblasts were linked to tumor-associated pathways in the NPC microenvironment

**Fig. 6:**
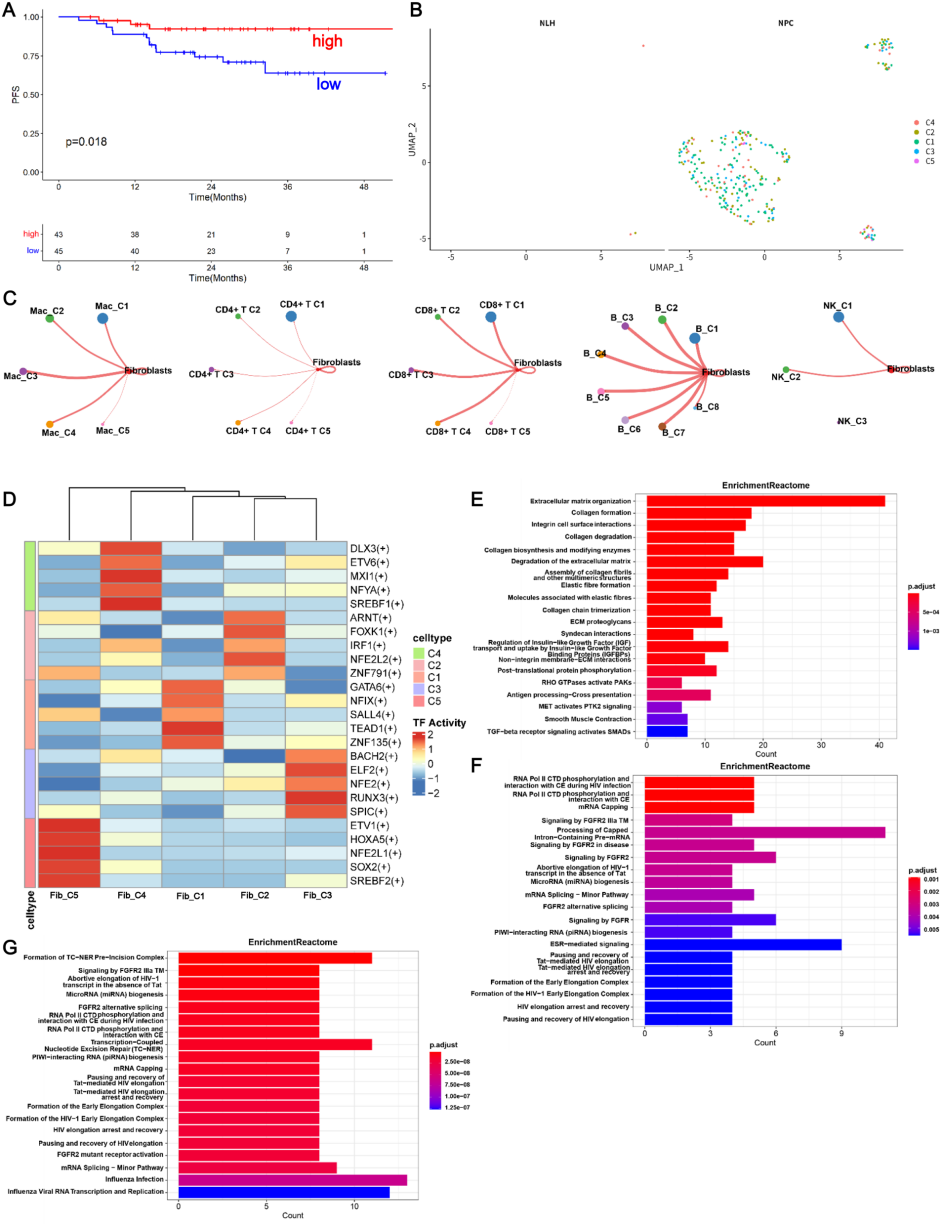
m7G-related fibroblasts participated in tumorigenesis. **A** Kaplan-Meier curve depicting the prognosis of fibroblast cells by survminer. **B** UMAP plot of the m7G-related fibroblasts. **C** Cell-cell communication analysis shows that fibroblasts interacted with other m7G-related immune cell clusters. **D** Transcription factor (TF) activities differed among the fibroblast clusters. **E**-**G** Reactome enrichment analysis demonstrates the activated signaling pathways and functions in the m7G-related Fib_C1 (**E**), Fib_C3 (**F**), and Fib_C4 (**G**)

Survival analysis based on the bulk RNA-seq data collected from GSE102349 showed that the high infiltration of fibroblasts indicated a poor prognosis in patients with NPC (Fig. 6A). Thus, the fibroblasts were clustered based on the expression of m7G regulators in the NPC samples to investigate that whether m7G modification affected fibroblasts in NPC TME. Remarkably, m7G-mediated fibroblasts primarily appeared in the NPC samples (Fig. 6B), which suggests the abnormal proliferation of fibroblasts in the NPC TME. Among the five fibroblast clusters, the m7G score of Fib_C4 was significantly higher in the NLH samples compared to the NPC samples (Supplementary Fig. S5A), and POLR2E was differentially expressed in Fib_C4 (Supplementary Fig. S5B). Cell–cell communication analysis revealed that fibroblasts mediated most interactions with other m7G-related immune cell clusters in the TME, including macrophages, T cells, B cells, and NK cells (Fig. 6C). SCENIC analysis showed that TF activities in each cluster were distinct (Fig. 6E). Notably, IRF1 [35], ETV6 [36], SREBF1 [32], RUNX3 [37], and TEAD1 [38], which are involved in NPC progression, were significantly activated in m7G-related fibroblasts (Fig. 6D). GSVA revealed that the Fib_C1-C4 enriched significant pathways including cell development (mitotic spindle, p53 pathway, PI3K/AKT/MTOR signaling, DNA repair, E2F targets, and G2M checkpoint) and cell metabolism (HEME metabolism, bile acid metabolism, fatty acid metabolism, adipogenesis, and glycolysis), which were inhibited in Fib_C5 (Supplementary Fig. S5C). Additionally, the enrichment map demonstrated that the Fib_C1 enriched pathways were related to extracellular matrix (ECM), fibres, collagen, and TGF-beta receptor signaling that activates SMADs (Fig. 6E). The fibroblast growth factor receptor (FGFR) downstream signaling pathway has been identified to play a critical role in the development of NPC [39]. Intriguingly, Fib_C3 (Fig. 6F) and Fib_C4 (Fig. 6G) were characterized by multiple FGFR2-associated signaling pathways, including signaling by FGFR2 IIIa TM, signaling by FGFR2 in disease, signaling by FGFR2, FGFR2 alternative splicing, and FGFR2 mutant receptor activation, which suggests an underlying correlation between fibroblasts and macrophages via FGFR2 signaling. Fib_C2 was also associated with ECM organization and signaling by FGFR2 (Supplementary Fig. S5D). Taken together, the biological pathways of tumors that participate in the progression of NPC are activated in m7G-related fibroblast clusters.

### M7G‑related TME cells interacted with each other via various ligand–receptor pairs and signaling pathways

**Fig. 7:**
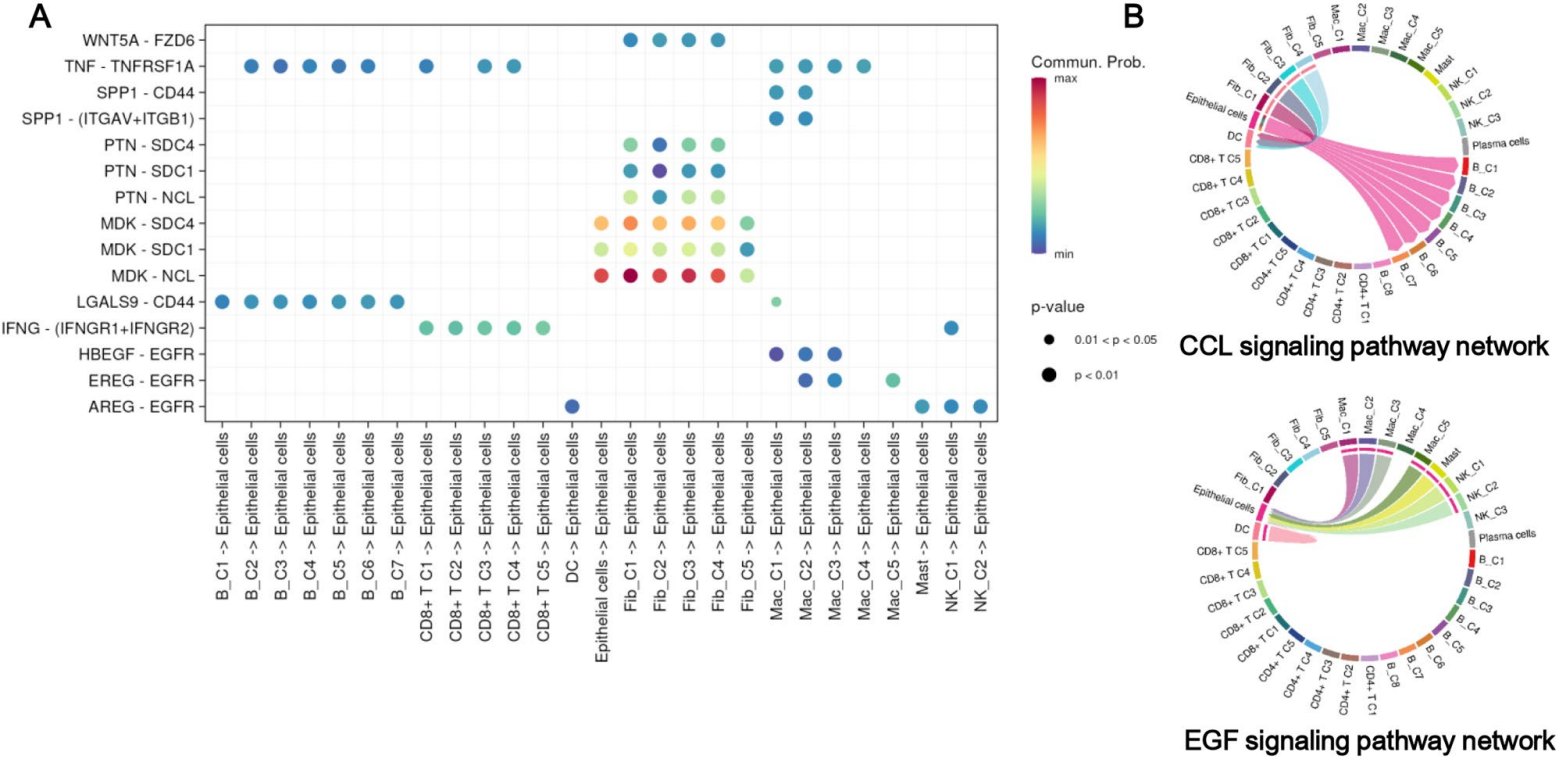
Correlation and cell–cell communication analysis of epithelial cells with other m7G-related tumor microenvironment (TME) cell clusters. **A** The significantly related ligand–receptor interactions between the main m7G-related clusters and epithelial cells. **B** Epithelial cells interacted with B cell clusters via the CCL signaling pathway, as well as with macrophage and natural killer (NK) cell clusters via the EGF signaling pathway

The communication between TME cells is important for the progression of NPC. As shown in Fig. 2C, there were various correlations between m7G-related TME cells, and epithelial cells were positively correlated with B_C3, CD4^+^ T_C4, and CD8^+^ T_C3. Through the cell–chat communication analysis, we found epithelial cells expressed a notable number of receptors, and we have the listed ligand–receptor pairs of intercellular communication between epithelial cells and other TME cell types (Fig. 7A). Most ligand–receptor pairs were identified between m7G-related fibroblasts and epithelial cells, such as WNT5A-FZD6, PTN-SDC4, PTN-SDC1, PTN-NCL, MDK-SDC4, MDK-SDC1, and MDK-NCL (Fig. 7A), in which MDK-NCL has been reported in the intercellular communication between fibroblasts and epithelial cells [40]. Galectin-9 encoded by *LGALS9* could bind to receptor CD44 to regulate anti-cancer immunity [41]. LGALS9-CD44 functioned as a ligand–receptor to maintain communication between m7G-related B cell clusters and epithelial cells, suggesting the potential role of m7G-mediated B cells in anticancer immunity. In addition, epithelial cells regulated m7G-related B cell clusters via the CCL signaling pathway, and were regulated by m7G-related macrophage clusters and NK cell clusters via the EGF signaling pathway (Fig. 7B). These interactions between m7G‑related immune cell clusters epithelial cells may further facilitate the progression of NPC.

## Discussion

NPC is a unique head and neck malignancy that originates from the nasopharynx [1]. The m7G modification is closely related to NPC, however, its regulatory roles in the NPC TME have not been reported. Here, we comprehensively summarized the landscape of m7G regulators in the NPC microenvironment using scRNA-seq data. The m7G-related immune cell clusters exhibited heterogeneous features in protumor activities, immune stimulation, and immunosuppression, and the m7G-related fibroblasts were enriched in tumor-associated pathways. Importantly, m7G-related TME cells interacted with each other via different ligand–receptor pairs and signaling pathways.

Our results provide the first comprehensive overview of m7G regulator expression within the NPC TME. Extensive analyses of m7G regulators have been demonstrated in multiple cancers. For example, m7G-related genes play important roles during the initiation and progression of colon cancer, where patients with a high-m7G score group had a higher survival rate than those with a low score, and are positively correlated with plasma B cells, CD8^+^ T cells, and regulatory T cells [42]. In lung adenocarcinoma, m7G-associated genes are associated with tumor immune infiltration in patients and are involved in TME regulation by stimulating various signaling pathways [43]. Consistently, different m7G modification patterns have different infiltration characteristics of TME cells in skin cutaneous melanoma, and high m7G scores are associated with high immune cell infiltration and stromal cell levels [44]. In our study, compared to NLH samples, the m7G scores of TME cells were significantly decreased in NPC samples, even though the proportion of TME cells was elevated. One study revealed two distinct m6A modification patterns in NPC that are consistent with immune-activated and immune-suppressed phenotypes, respectively [45]. The m7G-related genes are also involved in TME regulation via immunosuppression or immune activation. For example, METTL1 knockout in prostate cancer elicits a cytotoxic immune response and increases the infiltration of cytotoxic CD8^+^ T cells [46]. The samples related to m7G regulators in bladder cancer were positively correlated with many immune checkpoints [47]. Our results showed that the m7G-related immune cells in the NPC microenvironment exhibited phenotypes of immune activation and inhibition. Specifically, m7G-related CD4^+^ and CD8^+^ T cell clusters differentially expressed marker genes of immune co-inhibition and co-stimulation, and the pathways of immune co-inhibition and co-stimulation were enriched in the m7G-related macrophage clusters. RNA binding proteins including EIF3D, EIF4A1, POLR2L, POLR2G, and POLR2E were differentially expressed in the T cell and macrophage clusters. Aberrant expression of binding proteins is associated with the progression of several malignancies [48–50]. Importantly, EIF3D can facilitate the initiation of RAD51 translation, which ultimately leads to radiotherapy resistance in NPC [51]. We speculate that these RNA binding proteins may regulate the transcription of key genes through m7G methylation, thereby mediating the immune function of T cells and macrophages, which further affects the progression of NPC.

TLRs have been found to affect NPC progression by activating immune responses [52, 53], and TLRs can initiate downstream signaling cascades via MyD88 and IRAK4 [54]. In the macrophages, MyD88 deficiency (TLR2/4) and IRAK4 deficiency (TLR2/4) were enriched in the m7G-related macrophages, thus indicating that m7G-related macrophages were inhibited in the TLR2/4-mediated signaling pathway, which may contribute toward the development of NPC. Interestingly, m7G-mediated macrophage clusters were also involved in the regulation of immune responses in T cells through pathways of immune escape or immune activation, including PD-1 signaling, co-stimulation by the CD28 family, and TCR signaling, which were significantly enriched in the m7G-mediated macrophages. Thus, the effects of immunosuppression or immune activation of m7G-related immune cells in the TME are complex.

EIF4A1 was enriched in Mac_3 in the NPC samples, and the eukaryotic translation-related pathways were accumulated in Mac_C3. EIF4A1 binds to the m7G cap of mature mRNAs to launch the translation of open reading frames [55], and is located on chromosome 17p13, 667 bp upstream from the gene encoding the macrophage endosomal protein CD68 [56]. In this study, NPC increased EIF4A1 expression levels, and EIF4A1 was co-localized with CD86^+^ macrophages. CD86 is the macrophage M1 marker. We speculate that EIF4A1 may regulate macrophages via the transcriptional regulation of CD68, thereby causing macrophage M1 polarization. The epigenetic regulation of EIF4A1 transcripts plays an oncogenic role [57], and the prognosis of patients with cancer is associated with EIF4A1 [58, 59]. Currently, there is an increasing number of studies of EIF4A1 inhibitors for tumor treatment [55, 60, 61]. Here, EIF4A1 was negatively correlated with survival in patients with HNSCC, and EIF4A1 was co-localized with M1 macrophages. M1 macrophage polarization inhibits NPC cell growth and migration [62], thus suggesting a pivotal role of EIF4A1 in M1 macrophages during the development of NPC.

In addition, m7G regulator-mediated TME cells displayed tumor-associated signatures. In our study, the infiltrated levels of CD4^+^ T_C2, B_C3, and Mac_C3 in tumor samples were significantly higher than in normal samples. Correspondingly, the pathways involved in NPC development were activated in CD4^+^ T_C2 and B_C3, such as the TNFA signaling pathway via NF-κB [63] and MYC signaling [31]. PRMT1 contributes to the immune escape of cancer [64], and PRMT1 inhibition can activate the antitumor immunity [65]. B_C3 exhibited a high expression of PRMT1, which suggests a potential link between tumor-associated signatures of B cells and elevated PRMT1 levels.

A previous study demonstrated that m7G-related genes, METTL1/WDR4, can promote NPC growth and metastasis [66]. Similarly, the results of this study indicated that m7G regulator-mediated TME cells might contribute to tumorigenesis in NPC. The FGFR signaling pathway has been identified to play a critical role in the development of tumors, and FGFR-targeted therapies have emerged for the treatment of mammary tumors and intrahepatic cholangiocarcinoma [67, 68]. In our study, m7G-related fibroblast clusters significantly enriched the FGFR2 signaling pathways. One study reported that FGFR2 is overexpressed in cancer tissues from patients with NPC and multiple NPC cell lines, and FGFR2 silencing enhances the effect of cisplatin treatment [39]. We speculate that m7G-related genes are involved in the regulation of the TME by activating the FGFR2 signaling pathway in NPC. In addition, m7G-related fibroblasts exhibited characteristics that are associated with EMT, and EMT regulators (TWIST and SOX4) were specially activated in the m7G-related B cells. Moreover, cancer-associated KEGG pathways were enriched in m7G-related macrophages. These results confirm that the m7G modification is closely related to the NPC TME. In the intercellular communication results, m7G-related immune cells and m7G-related fibroblasts interacted with epithelial cells through different ligand–receptor pairs, which suggests m7G regulators can influence the EMT progression by regulating the interactions between TME cells and epithelial cells.

## Conclusion

In summary, our study reveals the levels and functional enrichment of TME cells in NPC under the regulation of m7G-related genes, and these TME cell clusters exhibit immunosuppressive, immune activation, and tumorigenesis characteristics. These results provided a novel perspective on NPC development and the potential therapy.

### Electronic supplementary material

Below is the link to the electronic supplementary material.

Supplementary Material 1

Supplementary Material 2

Supplementary Material 3

Supplementary Material 4

Supplementary Material 5

## Data Availability

The datasets supporting the conclusions of this article are available in the GEO repository, including GSE150825, GSE102349, and GSE68799 datasets.GSE150825 and its hyperlink to dataset https://www.ncbi.nlm.nih.gov/geo/query/acc.cgi?acc=GSE150825.GSE102349 and its hyperlink to dataset https://www.ncbi.nlm.nih.gov/geo/query/acc.cgi?acc=GSE102349.GSE68799 and its hyperlink to dataset https://www.ncbi.nlm.nih.gov/geo/query/acc.cgi?acc=GSE68799.

## Abbreviations

NPC: Nasopharyngeal Carcinoma
m7G: N7-methylguanosine
TME: Tumor Microenvironment
NLH: Nasopharyngeal Lymphatic Hyperplasia
NMF: Nonnegative Matrix Factorization
TAM: Tumor-Associated Macrophage
CAFs: Cancer-Associated Fibroblasts
tRNA: Transfer RNA
HCC: Hepatocellular Carcinoma
EMT: Epithelial-Mesenchymal Transition
scRNA-seq: Single-Cell Rna Sequencing
UMAP: Uniform Manifold Approximation And Projection
GSVA: Gene Set Variation Analysis
TFs: Transcription Factors
GEO: Gene Expression Omnibus
ECM: Extracellular Matrix
SCENIC: Single-cell Regulatory Network Inference And Clustering
NK: Natural Killer
DCs: Dendric Cells
HNSCC: Head and Neck squamous cell carcinoma
