# Supplementary figures and images for: Tumor-associated characteristics and immune dysregulation in nasopharyngeal carcinoma under the regulation of m7G-related tumor microenvironment cells
Source: World J Surg Oncol. 2024 Jun 25;22:166. doi: 10.1186/s12957-024-03441-2 (PMC11202337; doi:10.1186/s12957-024-03441-2)

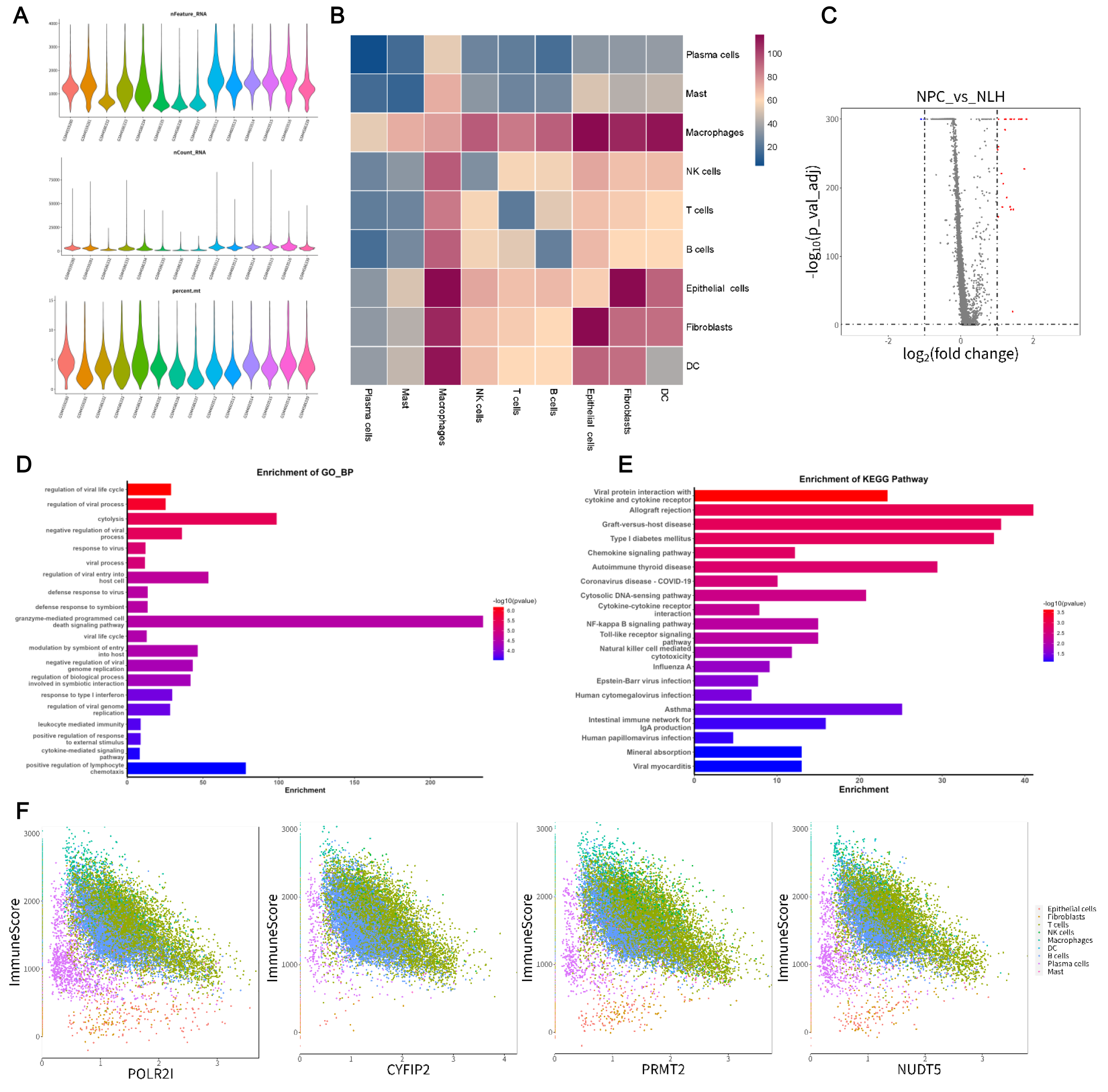

Supplement: Supplementary file 1 — Supplementary Material 1 [file 12957_2024_3441_MOESM1_ESM.tif]

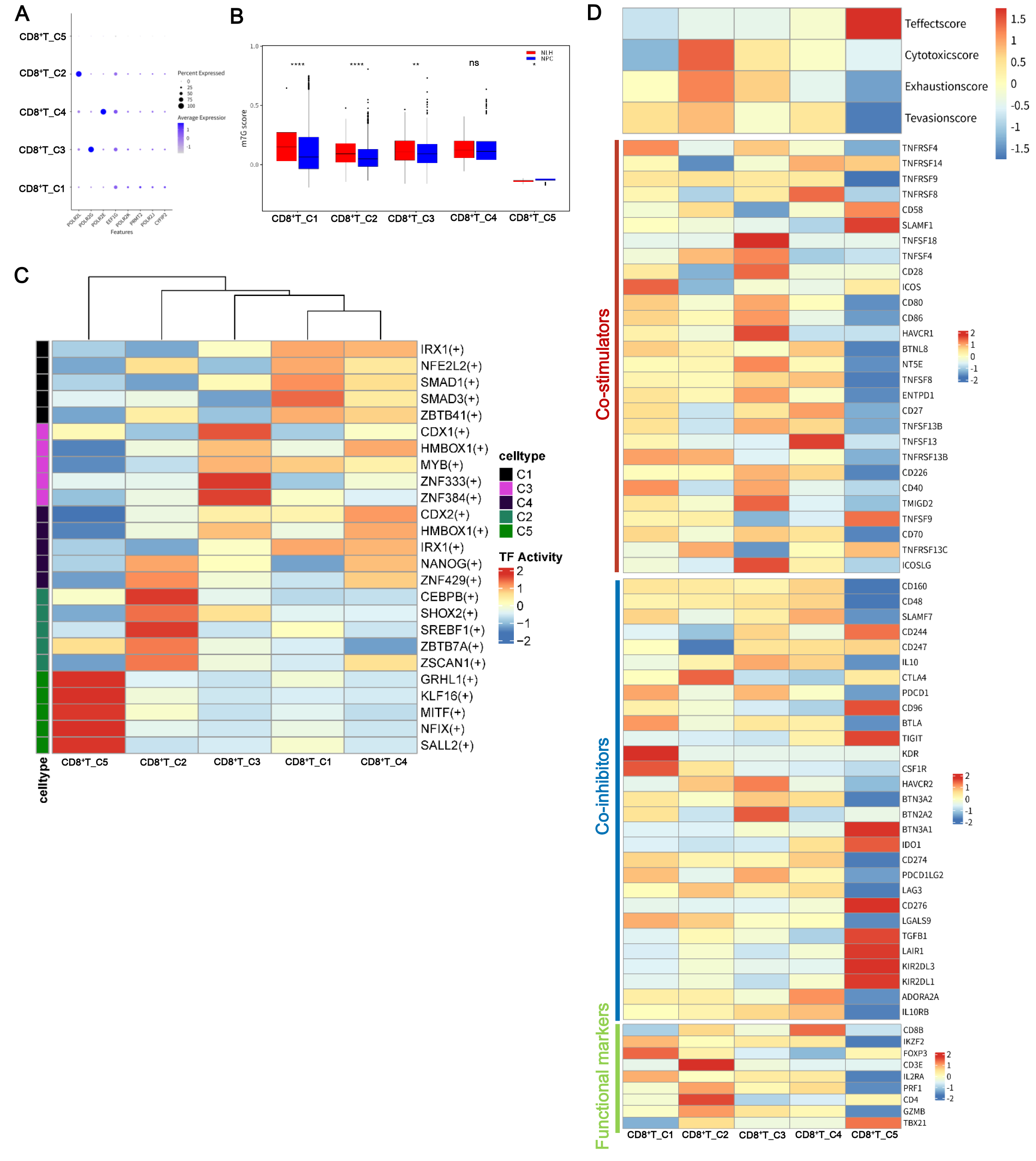

Supplement: Supplementary file 2 — Supplementary Material 2 [file 12957_2024_3441_MOESM2_ESM.tif]

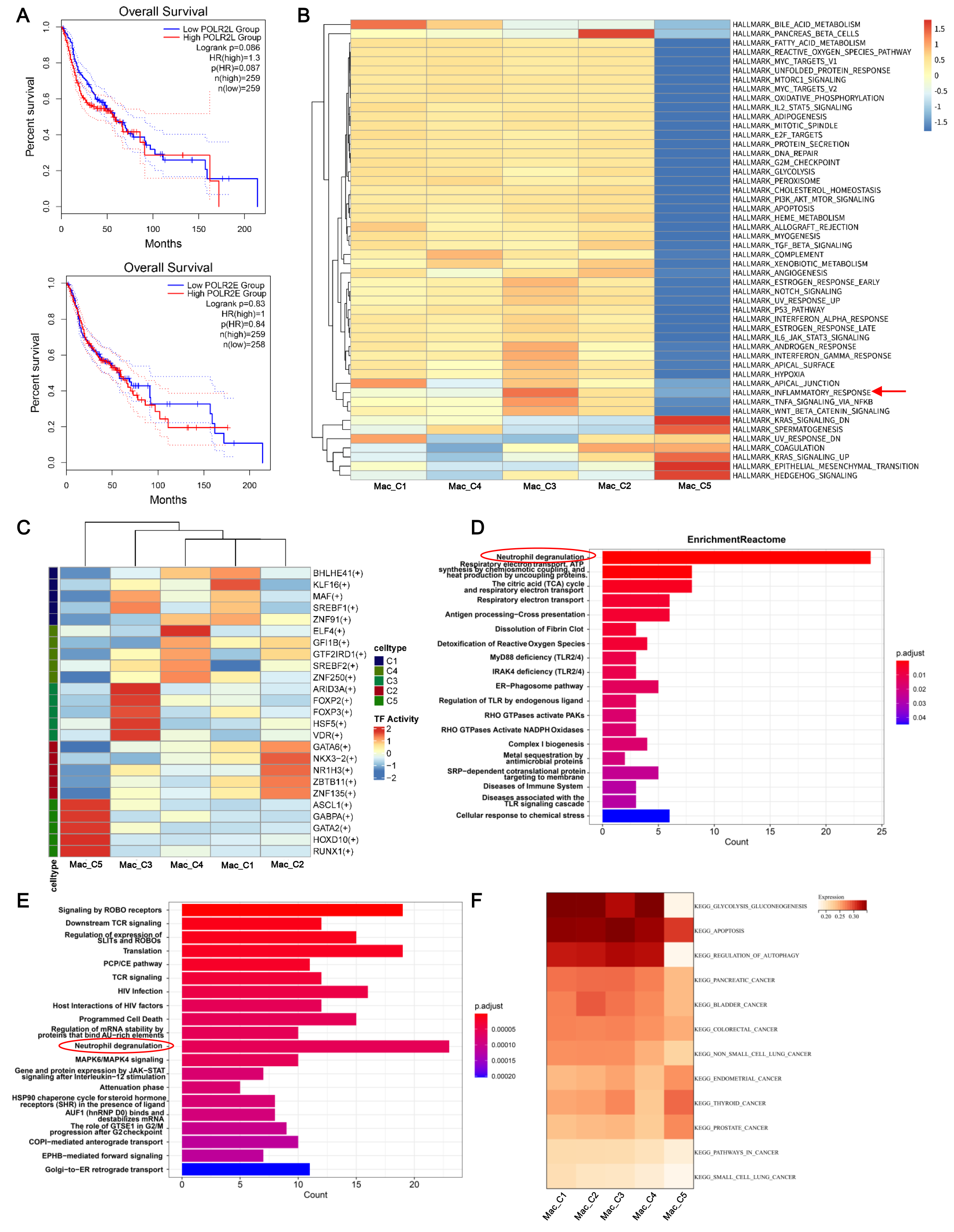

Supplement: Supplementary file 3 — Supplementary Material 3 [file 12957_2024_3441_MOESM3_ESM.tif]

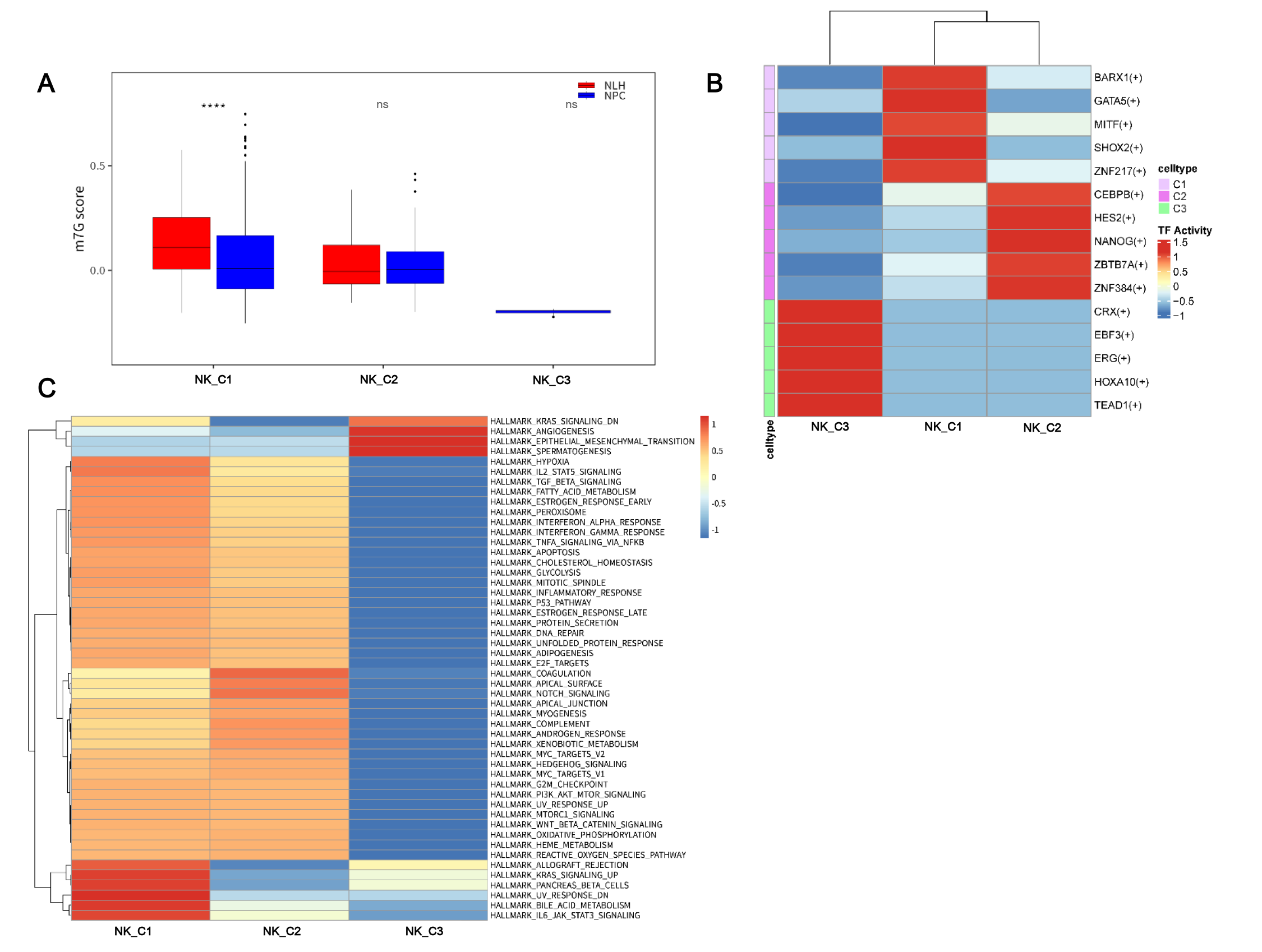

Supplement: Supplementary file 4 — Supplementary Material 4 [file 12957_2024_3441_MOESM4_ESM.tif]

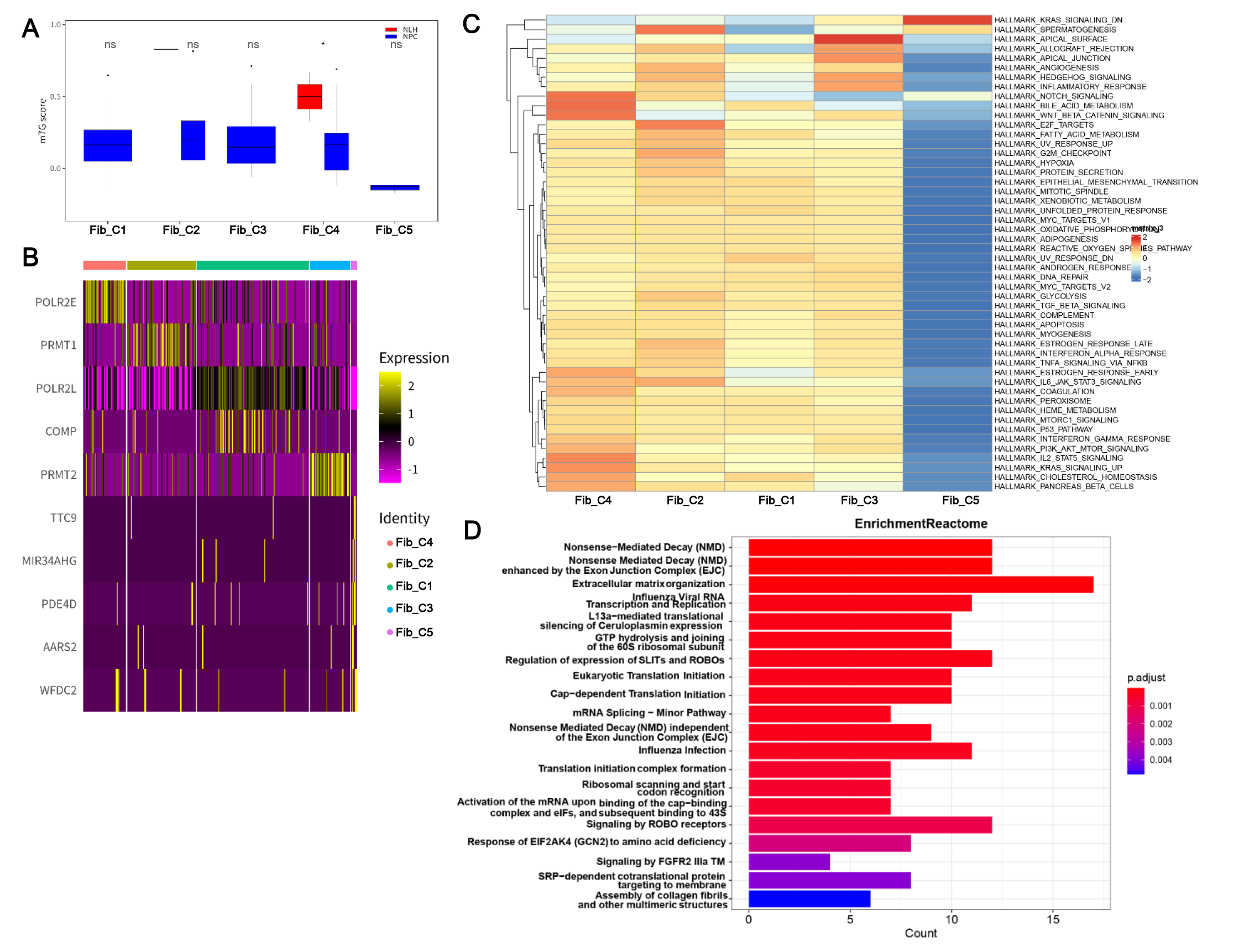

Supplement: Supplementary file 5 — Supplementary Material 5 [file 12957_2024_3441_MOESM5_ESM.tif]
